# Intestinal dysbacteriosis-induced IL-25 promotes development of HCC via alternative activation of macrophages in tumor microenvironment

**DOI:** 10.1186/s13046-019-1271-3

**Published:** 2019-07-11

**Authors:** Qiao Li, Lei Ma, Shunli Shen, Yu Guo, Qinghua Cao, Xiuqin Cai, Juan Feng, Yuan Yan, Tianyu Hu, Shiya Luo, Lin Zhou, Baogang Peng, Zhonghan Yang, Yunpeng Hua

**Affiliations:** 1 Department of Liver Surgery, The First Affiliated Hospital of Sun Yat-sen University, Guangzhou, Guangdong 510080 People’s Republic of China; 2 Department of Biochemistry, Zhongshan School of Medicine, Sun Yat-sen University, Guangzhou, Guangdong 510080 People’s Republic of China; 3 Cancer Center & Precision Medicine Institute, the First Affiliated Hospital of Sun Yat-sen University, Guangzhou, Guangdong 510080 People’s Republic of China; 4 Department of Pathology, the First Affiliated Hospital of Sun Yat-sen University, Guangzhou, Guangdong 510080 People’s Republic of China; 5 Department of Gastrointestinal Surgery, the First Affiliated Hospital of Sun Yat-sen University, Guangzhou, Guangdong 510080 People’s Republic of China; 6 School of Stomatology and Medicine, Foshan University, Foshan, Guangdong 528000 People’s Republic of China; 7 Department of Histology and Embryology, College of Basic Medicine, Southern Medical University, Guangzhou, Guangdong 510515 People’s Republic of China

**Keywords:** Hepatocellular carcinoma, Gut microbiota, Tumor microenvironment, Interleukin-25, Macrophages, Chemokine

## Abstract

**Background:**

Gut microbiota and the tumor microenvironment are thought to be critical factors that modulate the processes of liver diseases, including hepatocellular carcinoma (HCC). Interleukin-25 (IL-25) promotes type 2 immunity via alternative activation of macrophages, and is closely associated with inflammation-related diseases, even malignancies. However, it is not clear which role IL-25 plays in the development of HCC, and whether gut microbiota are involved.

**Methods:**

IL-25 was detected by ELISA, Western blotting (WB), and immunohistochemistry. Chemokines were measured by RT-qPCR and WB. After co-culture with IL-25-stimulated macrophages, the cell growth, migration, invasion and EMT marker of HCC cell lines (MHCC97L and HepG2) were evaluated by Brdu proliferation, Transwell assays and WB. An antibody neutralization assay of chemokine CXCL10 was performed to confirm its role in HCC development. Furthermore, the effects of IL-25 in HCC were investigated in vivo. Dysbiosis of gut microflora was induced by antibiotics (vancomycin, cefoperazone or combination of ampicillin, neomycin, metronidazole, and vancomycin). We used feces suspension to treat colonic epithelial NCM460 cells, and detected IL-25 and tuft cell marker DCLK1 using WB and immunofluorescence staining.

**Results:**

We found that the level of IL-25 was significantly elevated in HCC patients, and was negatively correlated with survival rate after hepatectomy. However, IL-25 did not directly promote the development of HCC cells. Then, we observed the significant positive correlation between IL-25 level and M2 percentage (CD206/CD68) in HCC tumors. In vitro and in vivo, IL-25 induced alternative activation of macrophages promoted HCC cell migration, invasion and tumorigenesis, increased the expression of vimentin, Snail and phospho-ERK, and decreased the expression of E-cadherin in HCC cells. After IL-25 treatment, chemokine CXCL10 was increased in macrophages. Neutralizing CXCL10 in macrophage-conditioned medium reversed the IL-25-mediated effect on HCC cells. Vancomycin-induced dysbiosis promoted the growth of orthotopic HCC homograft. Surprisedly, we found the hyperplasia of colonic epithelial tuft cells, from which more IL-25 was secreted .

**Conclusions:**

IL-25 promotes the progression of HCC through inducing alternative activation and CXCL10 secretion of macrophages in tumor microenvironment, and IL-25 secretion may partly result from hyperplastic epithelial tuft cells in colon, induced by gut microbiota dysbiosis.

**Electronic supplementary material:**

The online version of this article (10.1186/s13046-019-1271-3) contains supplementary material, which is available to authorized users.

## Background

Hepatocellular carcinoma (HCC) is the third leading cause of cancer-related death [1], despite recent advances in the diagnosis and treatment of HCC. Tumor progression with metastasis is the main cause of death in HCC patients; however, its underlying mechanisms are still unknown [2].

Recently, interest has grown in the cross-talk between tumor cells, the tumor microenvironment (TME), and gut microbiota. The gut microbiota influences whole host biology or ‘tumor macroenvironment’, and together with the TME modulates the processes of liver fibrosis, hepatocarcinogenesis, epithelial-to-mesenchymal transition (EMT), tumor invasion, and metastasis [2]. Tumor-associated macrophages (TAM) and their related inflammatory cytokines are considered important components of the tumor macro- and micro- environment, and are closely associated with the progression of HCC [3–5].

Interleukin (IL)-25 (also named IL-17E) is a member of the IL-17 cytokine gene family, and has been shown to play a critical role in the promotion of type 2 immunity, which is important against parasitic nematode infections [6–9]. The literatures have reported that IL-25 plays leading roles in fulminant hepatitis (FH), hepatic fibrosis and hepatic steatosis [10–12]. Our previous studies also showed that IL-25 reduced body weight gain and lipid accumulation by stimulating Type-2 macrophages (M2) polarization in nonalcoholic fatty liver disease (NAFLD) [13]. It is well-known that M2 macrophages, the most important TAMs, play an important role in immune suppression, and contribute greatly to HCC growth and metastasis [3, 4, 14]. However, the role of IL-25 in HCC development is not clear, and whether IL-25-induced M2 macrophages promote HCC invasion and metastasis is unknown.

In the present study, we firstly detected the expression of IL-25 and evaluated its relevance with prognosis in HCC patients. Then, we explored the mechanisms and the roles of IL-25 in the development of HCC, in particular, the cross talk between IL-25, macrophages and HCC. Intriguingly, several studies have reported that IL-25 is derived from intestinal epithelial cells [15–18]. We further probed the source of IL-25 and the mechanisms of gut dysbacteriosis promoting HCC.

## Methods

### Patients

All patients in this study were from The First Affiliated Hospital of Sun Yat-sen University. The tissue microarray consisted of 98 malignant liver tissues and 55 normal liver tissues. Malignant liver tissues were from patients with pathologically confirmed HCC after curative surgery. Normal liver tissues were from patients who underwent hepatectomy for hepatic hemangioma (HH). Blood and fresh tissue samples were collected from 5 HH patients and 10 patients with HCC. None of the individuals were positive for hepatitis C virus (HCV) or human immunodeficiency virus (HIV), consumed excessive alcohol, or received chemotherapy prior to sampling. All patients provided written informed consent before the study was initiated. This study was approved by the Ethics Review Committee of the First Affiliated Hospital of Sun Yat-sen University. All the data of human subjects are summarized in Additional file 1: Table S1.

### Immunohistochemistry and immunofluorescence

A tissue microarray and frozen sections were used to examine the level of IL-25 and number of M2 macrophages in normal liver tissue and tumor tissue by immunohistochemistry and immunofluorescence staining. The detailed techniques have been described in Additional file 1 and our previous study [13, 19]. IL-25 immunohistochemistry staining was scored by applying a semiquantitative immunoreactivity score (IRS) reported elsewhere [20]. The number of CD68 and C206 positive-stained cells was determined and quantified using the Image Scope positive pixel count algorithm (Aperio) [3]. We divided the 98 HCC patients into 2 groups according to the third quartile of the IL-25 scores or M2 percentage (CD206/CD68): low expression group (*n* = 70), high expression group (*n* = 28). The clinical characteristics of these two groups are summarized in Additional file 1: Table S2.

### Cell lines

The human HCC cell lines MHCC97L and HepG2, human colonic epithelial cell line NCM460, mouse HCC cell line H22, and Hepa1–6 cell line were maintained in Dulbecco’s modified Eagle’s medium (DMEM) with 10% fetal bovine serum (FBS) (Gibco by Life Technologies, Bleiswijk, the Netherlands). Human monocyte cell line THP-1 cells were cultured in RPMI 1640 with 10% FBS. Except Hepa1–6 cell line from American Type Culture Collection, the other cell lines were purchased from the Shanghai Cell Collection (Shanghai, China).

### Macrophage preparation and polarization

The protocols of M0 and M2 macrophage polarization from human monocyte cell line THP-1 cells were as described previously [3, 13]. Briefly, THP-1 cells were polarized to M0 or M2. THP-1 cells were treated with 200 ng/ml of phorbol 12-myristate13-acetate (PMA, Sigma-Aldrich, USA) for 24 h, washed, and then cultured for another 24 h to induce M0 cells. Subsequently, the cells were treated with 100 ng/ml of human recombinant IL-25 (BioLegend) for 48 h to induce M2 cells.

### Co-culture with macrophages and neutralization of CXCL10

To establish a cell co-culture system, 6 × 10^5^ THP-1 cells were seeded in 0.4 μm sized pores inserts (Corning, USA) and polarized into M2 macrophages. The culture medium was then replaced with RPMI 1640 medium without FBS and culture was continued for another 24 h. Inserts containing macrophages were transferred into 6-well cell culture plates seeded with MHCC97L or HepG2 cells (2.5 × 10^5^ cells per well) in advance. After co-cultured for 48 h, the HCC cells were detected by Brdu proliferation assays and Western blotting (WB).

To determine the role of CXCL10 produced by macrophages, 2.5 μg/ml CXCL10 neutralizing antibody (Abcam) was added to the culture medium to neutralize CXCL10 protein.

### Analysis of tumor cell proliferation, migration, invasion and apoptosis

After treatment with IL-25 or co-culture with IL-25-stimulated macrophages, the cell growth, migration and invasion of HCC cell lines (MHCC97L and HepG2) were evaluated by Cell Counting Kit-8 (CCK-8) (Dojindo, Japan), Brdu staining (Sigma-Aldrich) and Transwell assays. The cell apoptosis of HCC cell lines were evaluated after treatment with IL-25 using FITC Annexin V staining (BD Biosciences). Please see the Additional file 1 for details.

### ELISA, WB and real-time quantitative PCR (RT-qPCR)

IL-25 in serum and liver of HCC patients was detected by ELISA and WB. M2 macrophages were induced as described above, and the expression of chemokines were measured by RT-qPCR and WB. The primers used for the amplification of human genes were listed in Additional file 1: Table S3. RT-qPCR, ELISA and WB was performed as described previously [13, 21, 22].

### Animal models

All studies were conducted with the approval of the Institutional Animal Care and Use Committee (IACUC) of the First Affiliated Hospital of Sun Yat-sen University. An orthotopic-transplanted liver tumor model was created in BALB/c nude mice with portal venous injection of macrophages [3]. In addition, a subcutaneous implanted tumor model and the orthotopic-transplanted liver tumor model were prepared in C57BL/6 mice. Dysbiosis of gut microflora was induced by antibiotics (vancomycin, cefoperazone or combination of ampicillin, neomycin, metronidazole, and vancomycin) in C57BL/6 mice [23, 24]. Please see the Additional file 1 for details.

### 16S rRNA sequencing and analysis

Feces samples were collected from the orthotopic-transplanted liver tumor model of C57BL/6 mice with gut microbiota dysbiosis, and stored at − 80 °C until use. Total DNA in feces was isolated using the DNA extraction kit (Tiangen, China). The V3-V4 hypervariable regions of the bacteria 16S rRNA gene were amplified with primers 338F (5′-ACTCCTACGGGAGGCAGCAG-3′) and 806R (5′-GGACTACHVGGGTWTCTAAT-3′). PCR products were purified using the GeneJET Gel Extraction Kit (Thermo Scientific). Sequencing libraries were generated using Illumina TruSeq DNA PCR-Free Library Preparation Kit (Illumina, USA) following manufacturer’s recommendations and index codes were added. The sequence was performed by Illumina Hiseq platorm (Novogene Bioinformatics Technology Co., Ltd.). Sequences analysis was performed by Uparse software (Uparse v7.0.1001, http://drive5.com/uparse/). Sequences with ≥97% similarity were assigned to the same OTUs.

### Preparation and treatment of feces suspension

Feces samples were selected from normal mouse and antibiotics-treated mouse at random. Then, 100 mg feces was suspended in 5 ml sterile PBS. The suspension was filtered by 40 μm cell strainer (Corning) and 0.8 μm syringe filter (Corning) for obtaining feces suspensions with bacteria. The feces suspension with bacteria was then filtered by 0.22 μm syringe filter (Corning) for obtaining feces suspensions without bacteria. Colonic epithelial NCM460 cells were treated with 10% feces suspension in culture medium for 6 h, then the expression levels of IL-25 and DCLK1 were examined by immunofluorescence and WB.

### Statistical analysis

Data were presented as mean value ± standard deviation. Cell experiments were performed in triplicate, and at least 3 independent experiments were assessed. All analyses were performed with GraphPad Prism 5.0 and SPSS 19.0 software. Comparisons and correlations of quantitative data between 2 groups were performed by unpaired Student’s t test and chi-square test, respectively. Categorical data were analyzed by Fisher’s exact test. The overall survival (OS) and disease-free survival (DFS) rates were calculated by the Kaplan–Meier method, and survival curves were compared by log-rank test. Values of *p* < 0.05 were considered statistically significant.

## Results

### Increased IL-25 expression in HCC patients correlates with poor prognosis

**Fig. 1:**
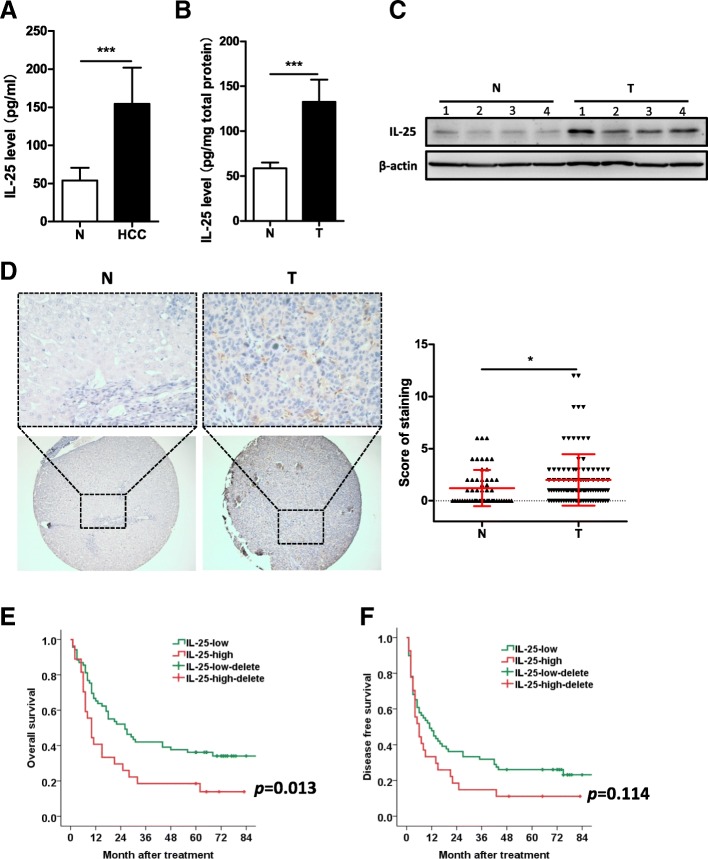
Overexpression of IL-25 is found in HCC patients, and predicts the poor prognosis. **a** Serum levels of IL-25 were detected by ELISA in hepatic hemangioma patients (*n* = 5) and HCC patients (*n* = 10). **b** Concentrations of IL-25 were detected by ELISA in tissue homogenates of normal liver tissues (*n* = 5) and HCC tumor tissues (*n* = 10). **c** Protein levels of IL-25 were detected by Western blotting in normal liver tissues (*n* = 4) and HCC tumor tissues (*n* = 4). **d** and **e** Immunohistochemistry (IHC) staining of IL-25 was performed in a tissue microarray consisted of 98 HCC tumor tissues and 55 normal liver tissues. **d** IL-25 representative IHC images (left) with statics (right) of the corresponding levels are shown. Bar, 20 μm (up), 100 μm (down). Overall survival (**e**) and disease free survival (**f**) curves of 98 HCC patients in correlation with intra-tumor IL-25 scores. The third quartile of the IL-25 scores was used as a cut-off value. The patients with HCC were divided into 2 groups according to the intra-tumor IL-25 score: low group (*n* = 70), high group (*n* = 28). The clinical characteristics of these two group are summarized in Additional file 1: Table S2. *p < 0.05, ***p < 0.001

The levels of IL-25 in serum and tissue were significantly increased in HCC patients (*n* = 10) as compared to non-cancer patients (HH) (*n* = 5) (Fig. 1a, b). Using WB, we similarly found that IL-25 protein was overexpressed in HCC tissue, compared with normal liver tissue (Fig. 1c). In addition, IL-25 immunostaining of HCC tumor tissue was significantly stronger than that of normal liver tissue of HH patients (Fig. 1d).

High expression of IL-25 in HCC patients was closely correlated with poor survival after resection (Fig. 1e, f). The 3-year OS rate of the IL-25-high-group (*n* = 28) (18.5%) was significantly lower than that of the IL-25-low-group (*n* = 70) (42%) (*p* = 0.013) (Fig. 1e). The 3-year DFS rate of IL-25-high-group (14.8%) was also lower than that of the IL-25-low-group (31.9%), however, their difference was not statistically significant (*p* = 0.114) (Fig. 1f). These findings indicate that a high level of IL-25 expression is associated with the development and poor prognosis of HCC.

### IL-25 promotes the migration and tumorigenesis of HCC cells via facilitating the M2-like phenotype

**Fig. 2:**
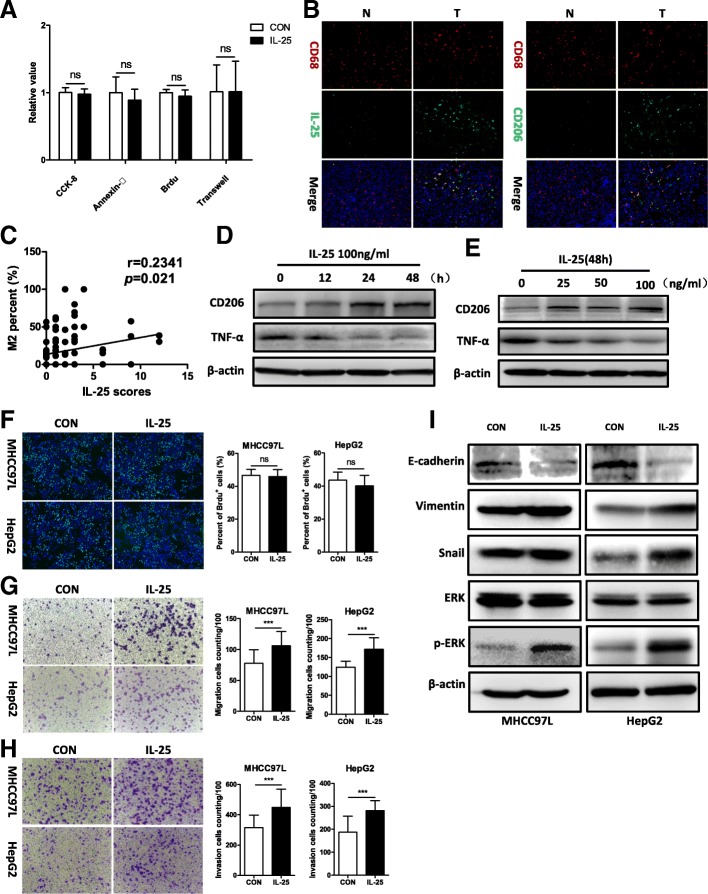
IL-25 activates alternative macrophages (M2), which promote HCC cell migration and invasion. **a** IL-25 was used to directly treated HCC cells in vitro, the relative value of CCK-8, Annexin-V, Brdu, Transwell experiment. (Please see results in Additional file 1: Figure S1A-D). **b** Immunofluorescent staining was performed in normal liver tissues (*n* = 55) and HCC tumor tissues (*n* = 98). Representative immunofluorescence images. Bar, 50 μm. **c** Immunohistochemistry staining was performed in a HCC tissue microarray (*n* = 98). Correlation between M2 macrophage (CD206/CD68) percentage and IL-25 scores. CD206 is an M2 macrophage marker, and CD68 is macrophage marker. **d** and **e** Macrophages (derived from THP-1) were treated with IL-25 in a time- and concentration-dependent manner. Tumor necrosis factor-α (TNF-α) and CD206 were examined by Western blotting. **f** and **i** HCC cells co-cultured with M0 or M2 macrophages. **f** Cell growth was determined with the Brdu kit. Statistical data were shown at the right. Bar, 100 μm. HCC cells migration (**g**) and invasion (**h**) were determined by Transwell assay. Statistical data were shown at the right. Bar, 100 μm. **i** Mesenchymal maker vimentin, EMT regulator Snail, epithelial marker E-cadherin, extracellular signal-regulated kinase (ERK), and p-ERK were detected by Western blotting. ***p < 0.001, ns, no significance

To determine if IL-25 promotes HCC development, and if so by what mechanism, we treated HCC cells (MHCC97L cells and HepG2 cells) with recombinant human IL-25, and found that IL-25 did not remarkably promote the growth and migration of HCC cells (Fig. 2a, Additional file 1: Figure S1A, C and D). In addition, IL-25 did not influence on the apoptosis of HCC cells (Fig. 2a, Additional file 1: Figure S1B). Furthermore, we observed the tumorigenesis of Hepa1–6 subcutaneous implanted cells in C57BL/6 mice (Additional file 1: Figure S1E). The weight of tumors in the IL-25 group (4216 ± 2165 mg) was more than the control group (3548 ± 2012 mg), but there was no significant difference between them (*p* = 0.637, Additional file 1: Figure S1E). These results indicate that IL-25 may not directly impact the proliferation and invasion of HCC cells.

Our previous studies and other literature have indicated that IL-25 can target and deliver negative signals to macrophages, induce macrophages to an M2-like phenotype, and suppress the production of proinflammatory cytokines [12, 13]. Furthermore, intra-tumor macrophages with an M2 phenotype (TAM) are correlated with poor prognosis in numerous malignancies [25]. In our study, the intensity of IL-25 immunofluorescent staining of HCC tumor tissue was positively correlated with the intensity of CD206 staining (M2 marker) (Fig. 2b). Correlation analysis showed that there was significant positive correlation between IL-25 and M2 percentage (CD206/CD68) in HCC tissue (*r* = 0.2341, *p* = 0.021) (Fig. 2c). In addition, the OS and DFS of the M2-high group were lower than that of the M2-low group, although the differences did not reach statistical significance (*p* = 0.373 and 0.726, respectively) (Additional file 1: Figure S2). Additional cells experiment showed that IL-25 stimulated THP-1-derived macrophages to transform into the M2 phenotype, with the M2 marker CD206 increased and the M1 marker TNF-α decreased (Fig. 2d, e). In addition, the effects of IL-25 were time- and concentration-dependent (Fig. 2d, e). We thus hypothesized that IL-25 promoted HCC cell growth or metastasis via inducing M2 macrophages.

To test this hypothesis, in vitro studies were conducted by co-culturing HCC cells with THP-1-derived macrophages (M2 or M0) induced by PMA (100 ng/ml) with or without IL-25 (100 ng/ml). Interestingly, the cell proliferation rate of HCC cells (MHCC97L cells and HepG2 cells) after co-culture with M2 macrophages was not increased, compared with that of the control (HCC cells co-cultured with M0 macrophages) (Fig. 2f). However, we found that the abilities of migration and invasiveness of HCC cells (MHCC97L cells and HepG2 cells) were significantly increased after co-culture with M2 macrophages, compared with those of the control group (Fig. 2g, h). At the same time, the mesenchymal maker vimentin and the EMT regulator Snail were significantly upregulated, while the epithelial marker E-cadherin was downregulated in HCC cells (MHCC97L, HepG2) co-cultured with M2 macrophages (Fig. 2i).

It is well known that the phosphorylation of extracellular signal-regulated kinase (ERK) promotes tumor EMT program [26–28]. We observed that phosphorylated ERK in HCC cells (MHCC97L, HepG2) was greatly overexpressed after co-culture with IL-25-induced M2 cells (Fig. 2i). Taken together, our data suggest that IL-25 promotes HCC cell migration and invasion via facilitating the M2-like phenotype.

### CXCL10, secreted by IL-25-induced activated M2 macrophages, is the key chemokine that promotes HCC development

**Fig. 3:**
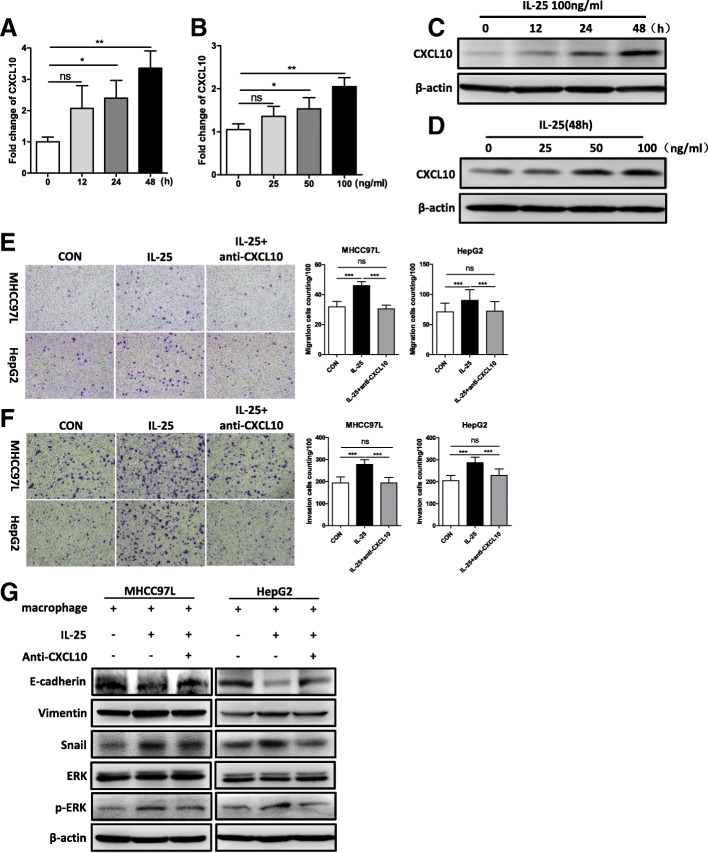
IL-25-induced M2 macrophages promote the EMT process in HCC cells via CXCL10. **a**-**d** Macrophages (derived from THP-1) were treated with IL-25 in a time- and concentration-dependent manner. **a** and **b** CXCL10 gene expression was quantified by RT-qPCR. **c** and **d** CXCL10 protein level was determined by Western blotting. **e**-**g** HCC cells were co-cultured with M0 or M2 macrophages. Then, anti-CXCL10 antibody was added to the M2 macrophage culture medium to neutralize CXCL10 protein. Migration (**e**) and invasion (**f**) of HCC cells were determined by Transwell assay. Statistical data were shown at the right. Bar, 100 μm. **g** Mesenchymal maker vimentin, EMT regulator Snail, epithelial marker E-cadherin, extracellular signal-regulated kinase (ERK), and p-ERK were detected by Western blotting. *p < 0.05, **p < 0.01, ***p < 0.001, ns, no significance

Type-2 macrophages (M2), as the most important TAM, always depend on secreting chemokines to promote tumor metastasis [3, 4]. Given that IL-25 promoted HCC cell migration via activated M2 macrophages, we postulated that the effect of M2 macrophages on HCC development might be the result of secretion of certain chemokines. Thus, we detected a series of tumor metastasis-related chemokines by RT-qPCR, which included CXCL1, CCL2, CCL5, CXCL8, CXCL9, CXCL10, CXCL12, CXCL15, CCL19, CCL20, CX3CL1, CCL17 and CCL22 [3, 4, 29–31]. Among them, the mRNA expressions of CXCL1, CCL2, CXCL10, and CCL17 were remarkably increased in IL-25-induced M2 macrophages, as compared with the control group (Additional file 1: Figure S3A). Furthermore, the level of CXCL10 mRNA was significantly elevated after IL-25 treatment in a time- and dose-dependent manner (Fig. 3a, b, Additional file 1: Figure S3B, C). Western blotting also demonstrated that CXCL10 protein was upregulated in the macrophages after IL-25 treatment in a time- and concentration-dependent manner (Fig. 3c, d). Taken together, these data suggest that CXCL10 is an important chemokine secreted by IL-25-induced M2 macrophages.

**Fig. 4:**
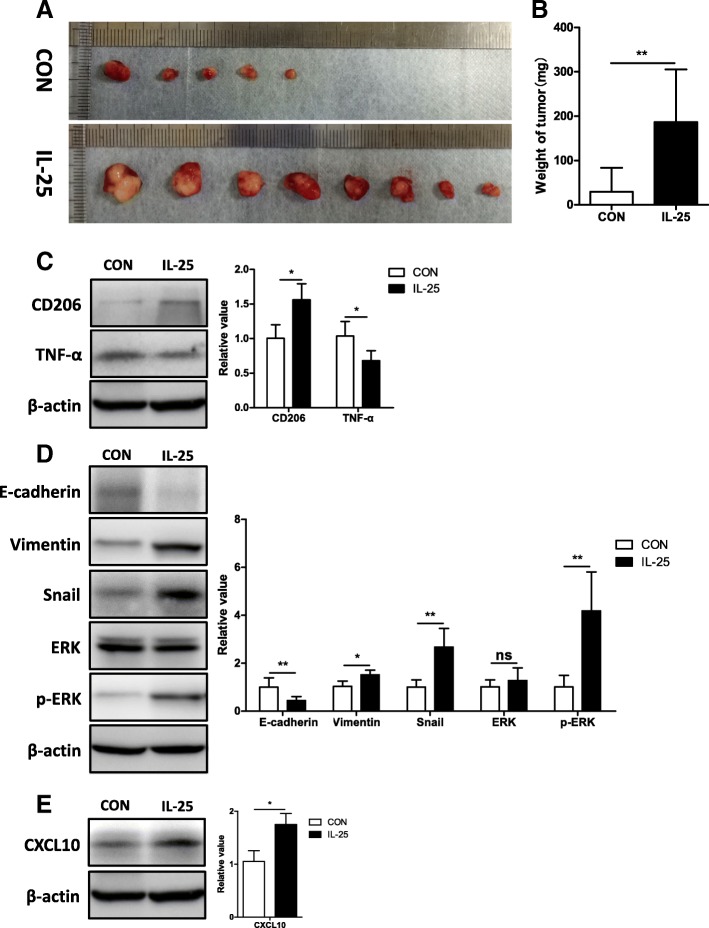
IL-25-induced M2 macrophages promote tumorigenesis and EMT of HCC cells in vivo. **a** Images of tumors from each group. **b** Tumor weight at the time of sacrifice. **c** Tumor necrosis factor-α (TNF-α) and CD206 in the tumor tissue of each group were detected by Western blotting. Statistical data were shown at the right. **d** Mesenchymal maker vimentin, EMT regulator Snail, epithelial marker E-cadherin, extracellular signal-regulated kinase (ERK), and p-ERK were detected in the tumor tissue of each group by Western blotting. Statistical data were shown at the right. **e** Chemokine CXCL10 was detected in the tumor tissue of each group by Western blotting. Statistical data were shown at the right. *p < 0.05, **p < 0.01, ns, no significance

To further confirm the role of CXCL10 on the migration and invasion of HCC cells, we used anti-CXCL10 antibody to neutralize CXCL10 in IL-25-induced M2 conditioned medium. The results showed that anti-CXCL10 antibody significantly decreased the migration and invasion of HCC cells (MHCC97L, HepG2) (Fig. 3e, f). Likewise, anti-CXCL10 antibody also decreased the levels of vimentin, Snail, and phosphorylation of ERK, but increased E-cadherin expression in MHCC97L and HepG2 cells with M2 conditioned medium (Fig. 4g). These results further support the idea that IL-25-induced M2 macrophages promote the EMT process in HCC cells via secreting CXCL10.

### IL-25-induced M2 macrophages promote tumorigenesis and EMT of HCC in vivo

To further confirm the role of IL-25-induced M2 macrophages on HCC cells in vivo, we established an orthotopic-transplanted liver tumor model in BALB/c nude mice with portal venous injection of macrophages. We found that the tumor formation rate in the group with IL-25-induced M2 macrophages was higher than that in the group with M0 macrophage (8/8 vs. 5/8, *p* = 0.06) (Fig. 4a). Similarly, the weight of tumors in the group with M2 macrophages (186.9 mg ± 118.7 mg) was significantly higher than that in the group with M0 macrophages (29.38 mg ± 54.28 mg) (*p* = 0.027) (Fig. 4a, b). Upregulation of the M2 marker CD206 and downregulation of M1 maker TNF-α were found in the group with M2 macrophages, as compared with the group with M0 macrophages (Fig. 4c). Consistent with these findings, the relative levels of vimentin, Snail, and phosphorylated ERK were significantly upregulated, and E-cadherin was significantly downregulated in the group with M2 macrophages (Fig. 4d). In addition, the level of CXCL10 was significantly increased in M2-treated group (Fig. 4e). These data further support that IL-25-induced M2 macrophages promote tumorigenesis and EMT of HCC cells, which might be closely related with CXCL10 secretion and ERK phosphorylation.

### Dysbiosis of gut microbiota results in hyperplasia of tuft cells and secretion of IL-25

**Fig. 5:**
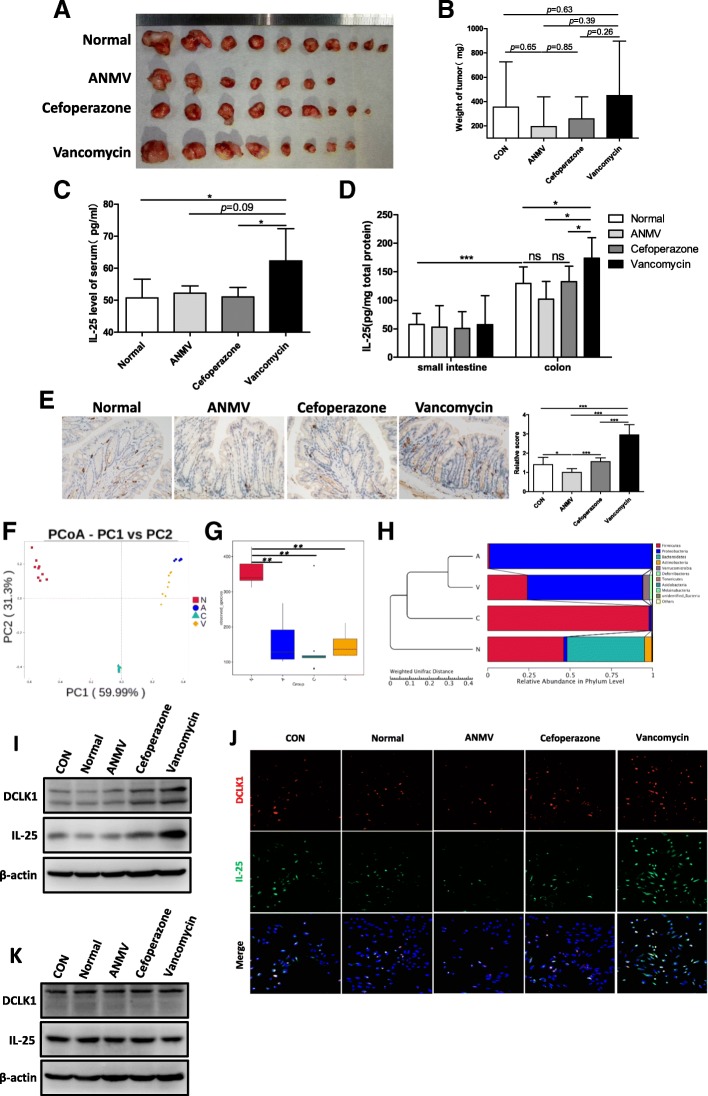
Gut bacterial dysbiosis promotes hyperplasia of tuft cells and secretion of IL-25. **a**-**e** An orthotopic C57BL/6 mice hepatic tumor model with gut microflora dysbiosis was prepared as described in the methods. **a** Images of tumors from each group. **b** Tumor weight at the time of sacrifice. **c** Serum level of IL-25 was detected by ELISA in each group. **d** Concentrations of IL-25 in small intestine and colon tissue homogenates were detected by ELISA. **e** Tuft cell marker DCLK1 representative IHC images. Statistical data were shown at the right. Bar, 20 μm. **f**-**h** 16S rRNA sequencing and analysis of feces gut microbiota of mice. N, normal control group. A, combination antibiotics group (ANMV). C, cefoperazone group. V, vancomycin group. **f** PCoA score based on weighted unifrac metrics was different in each group. **g** Observed bacterial species’ richness in feces samples from each group. *p* < 0.05 by Wilcoxon rank-sum test. **h** Hierarchical clustering of each group using Bray-Curtis dissimilarity indices at the phylum level by the weighted unifrac distances. **i** and **j** Feces suspensions with bacteria from the above groups were used to treat colonic epithelial NCM460 cells. **i** Western blotting was performed to determine DCLK1 and IL-25 levels. **j** Immunofluorescence staining was used to detect DCLK1 (red) and IL-25 (green) in slides embedded with NCM460 cells. Representative immunofluorescence images. Bar, 50 μm. **k** Feces suspensions without bacteria from the above groups were used to treat colonic epithelial NCM460 cells. Western blotting was performed to detect DCLK1 and IL-25 levels. *p < 0.05, **p < 0.01, ***p < 0.001, ns, no significance

The above data suggests that IL-25 is increased in HCC patients, is associated with poor prognosis, and that IL-25 promotes EMT and migration of HCC cells via activating M2 macrophages and promoting the secretion of CXCL10. However, it is not well-known where IL-25 is produced. Many studies have indicated that IL-25 is derived from intestinal epithelial cells, in particular Tuft cells [15–18]. Moreover, gut microbiota has been shown to play an important role in many diseases, especially in liver diseases and HCC [32]. Thus, we postulated that the dysbiosis of gut microbiota could impact tuft cells or induce the production of IL-25, and finally promote the development of HCC. In our study, dysbiosis of gut microbiota in C57BL/6 mice was produced using antibiotics in drinking water [23, 24]. Then, orthotopic HCC homografts were performed using the H22 mouse HCC cell line. The results showed that tumor size, tumor weight, and tumor formation rate in the vancomycin group (for killing Gram-positive bacteria), were greatest, compared with those in the normal control group, combination antibiotics group (ANMV), and cefoperazone group (Fig. 5a, b). Regretably, the differences were not statistically significant, probably because the sample size was relatively small. Likewise, the level of IL-25 in serum and colon tissue was significantly increased in the vancomycin group (Fig. 5c, d). Consistently, immunostaining results showed that the tuft cell marker DCLK1 in the colon was highly expressed in the vancomycin group, while DCLK1 in the ANMV group was most lowly expressed (Fig. 5e).

Simultaneously, 16S rRNA sequencing of feces gut microbiota of mice from the above groups was examined, then the difference of gut microbiota within the individual groups was analyzed by principle coordinates analysis (PCoA). The results suggested that the treatment of different antibiotics brought about the complete different bacterial community composition (Fig. 5f). The number of observed species in gut microbiota of mice was remarkably lowed after antibiotics treatment, compared with that in the normal control group (Fig. 5g). The gut microbiota in ANMV group mainly consisted of Proteobacteria, and they in cefoperazone group mainly consisted of Firmicutes, while they in vancomycin group showed the increased levels of Proteobacteria, Verrucomicrobia and Deferribacteres, as well as the decreased levels of Firmicutes and Bacteroidetes, compared to normal control group (Fig. 5h).

To evaluate the role of dysbacteriosis in the production of IL-25, feces suspensions with bacteria from the above groups were used to treat colonic epithelial NCM460 cells. As shown in Fig. 5i, the feces suspension with bacteria from vancomycin-treated mice enhanced the expression of the tuft cell marker DCLK1 and the secretion of IL-25. Similar results were observed in the immunofluorescence assay (Fig. 5j). However, the feces suspension without bacteria did not produce similar results (Fig. 5k). Taken together, our results suggest that vancomycin-induced dysbacteriosis induces hyperplasia of colonic epithelial tuft cells and facilitate the secretion of IL-25 from tuft cells, which may be closely related with HCC development.

## Discussion

As an anti-inflammatory cytokine, IL-25 promotes type 2 cytokine-dependent immunity, and limits the production of proinflammatory cytokines through inhibiting expression of type 1 cytokines. The deregulation of IL-25 has been found in many inflammation-related diseases, including helminthes parasitic infections, inflammatory bowel disease, asthma, fulminant hepatitis, and NAFLD [6, 10, 12, 33]. Simultaneously, IL-25 also plays an important role in several human cancers [34–38]. But, it is not known if IL-25 affects the development of HCC.

We found that IL-25 was significantly elevated in the serum and tissue of HCC patients, and high IL-25 expression in HCC tissue was negatively correlated with survival rate after radical hepatectomy. So we first indicated that IL-25 may be associated with a poor prognosis of HCC patients. Previous studies suggested that IL-25 played a direct role in cancer cells to affect the development of breast cancer [35–37]. Surprisingly, our results indicated that IL-25 was not found to directly impact the growth, apoptosis, or migration of HCC cells.

Our previous studies had clearly indicated that M2 macrophages induced by IL-25 alleviated obesity and NAFLD [13]. Similarly, Wang et al. reported that IL-25 induces liver macrophages to the M2 phenotype, negatively regulates the proinflammatory immune microenvironment, and ameliorates HDF-induced hepatic steatosis [12]. Rizzo et al. reported that IL-25-induced alternatively activated macrophages inhibited colitis [39]. In addition, Zhujun Jiang et al. reported that inhibition of IL-25 resulted in decreased type 2 T cells and macrophages in the primary tumor microenvironments, and enhanced breast tumor invasion and subsequent metastasis to the lung [34]. These findings suggest that macrophages are a key target of IL-25, and activation to the M2 phenotype may be the main pathway by which IL-25 promotes the development of HCC.

Originally, M2 phenotype macrophages were thought to be involved in the wound healing processes via extracellular matrix remodeling, angiogenesis, and immunosuppression [40]. However, numerous studies also observed that TAMs exhibiting the M2 phenotype were associated with a poor prognosis in many malignancies [25]. With respect to HCC, Yeung et al. reported that high M2-specific CD163 levels predicted a poor prognosis, and were related with increased tumor number and vascular invasion [3]. In addition, M2 macrophages promoted the growth and metastasis of HCC cells in vivo and in vitro [3, 4, 14]. Our study similarly showed that the number M2 macrophages (marked by CD206) was greater in HCC patients than in individuals without cancer (HH), and the percentage of M2 macrophages in HCC tissue was negatively correlated with DFS and OS of HCC patients with hepatectomy. Intriguingly, we further found that there was a significantly positive correlation between IL-25 level and M2 percentage (CD206/CD68) in HCC tumors. So we presumed that IL-25 facilitated the tumorigenesis and development of HCC via activating the M2 phenotype of macrophages.

Our results indicated that IL-25-induced M2 macrophages, rather than M0 macrophages, promoted the migration and invasion of HCC cells in vitro, and the formation and size of orthotopic-transplanted liver tumors in vivo. Moreover, the elevated level of EMT–related markers (vimentin, Snail, and phosphorylated ERK) in HCC cells supported that IL-25-induced M2 macrophages could promote HCC development via activating the EMT of HCC cells. However, it is not clear how IL-25-induced M2 macrophages activate the EMT of HCC cells?

Recently, the important role of chemokines in promoting tumor development has been highlighted in different cancers, including HCC [3, 4, 31]. Chemokines, released by tumor-associated host cells and cancer cells, can recruit and activate different cell types to control the balance between anti-tumor and pro-tumor responses in the TME [31]. So we detected the levels of chemokines in IL-25-induced M2 macrophages, and found that the level of CXCL10 in macrophages was markedly increased during treatment with IL-25 in a time- and concentration-dependent manner. Moreover, neutralization of CXCL10 with anti-CXCL10 antibody inhibited the effect of M2 macrophages promoting tumor migration. Simultaneously, the levels of E-cadherin, vimentin, Snail, and phospho-ERK were also reversed with the treatment of anti-CXCL10 antibody. These findings indicate that IL-25-induced M2 macrophages might enhance the progression of HCC cells by releasing CXCL10 and activating the EMT-related pathway.

Then, we sought to identify the source of increased IL-25. Studies have indicated that intestinal epithelial tuft cells produce IL-25 after helminth infection [15, 16]. And previous studies also have reported that the expression of IL-25 by intestinal epithelial cells was dependent on the presence of commensal bacteria [17, 18]. In view of the role of gut microbiota in HCC development, we hypothesized that dysbiosis of gut microbiota induced hyperplasia of intestinal epithelial tuft cells and the production of IL-25, which then promoted the tumorigenesis and metastasis of HCC via active M2 macrophages. Our results showed that a vancomycin-induced intestinal flora disturbance promoted the formation and growth of orthotopic HCC xenografts. In addition, hyperplasia of colonic tuft cells was observed after vancomycin treatment, along with an increase of IL-25 expression. Moreover, expression of the tuft cell marker DCLK1 and IL-25 was increased in colonic epithelial NCM460 cells after treatment with a feces suspension with bacteria from vancomycin-treated mice, but not a feces suspension without bacteria. The analysis of feces gut microbiota from mice treated by antibiotics further confirmed that the vancomycin treatment increased levels of Gram-negative bacteria in feces, such as Proteobacteria, Verrucomicrobia and Deferribacteres, but decreased levels of Gram-positive bacteria, such as Firmicutes. So we deduce that gut bacterial dysbiosis with enrichment of Gram-negative bacteria, rather than bacterial metabolites, results in the hyperplasia of colonic epithelial tuft cells with an increase of IL-25 secretion.

## Conclusions

**Fig. 6:**
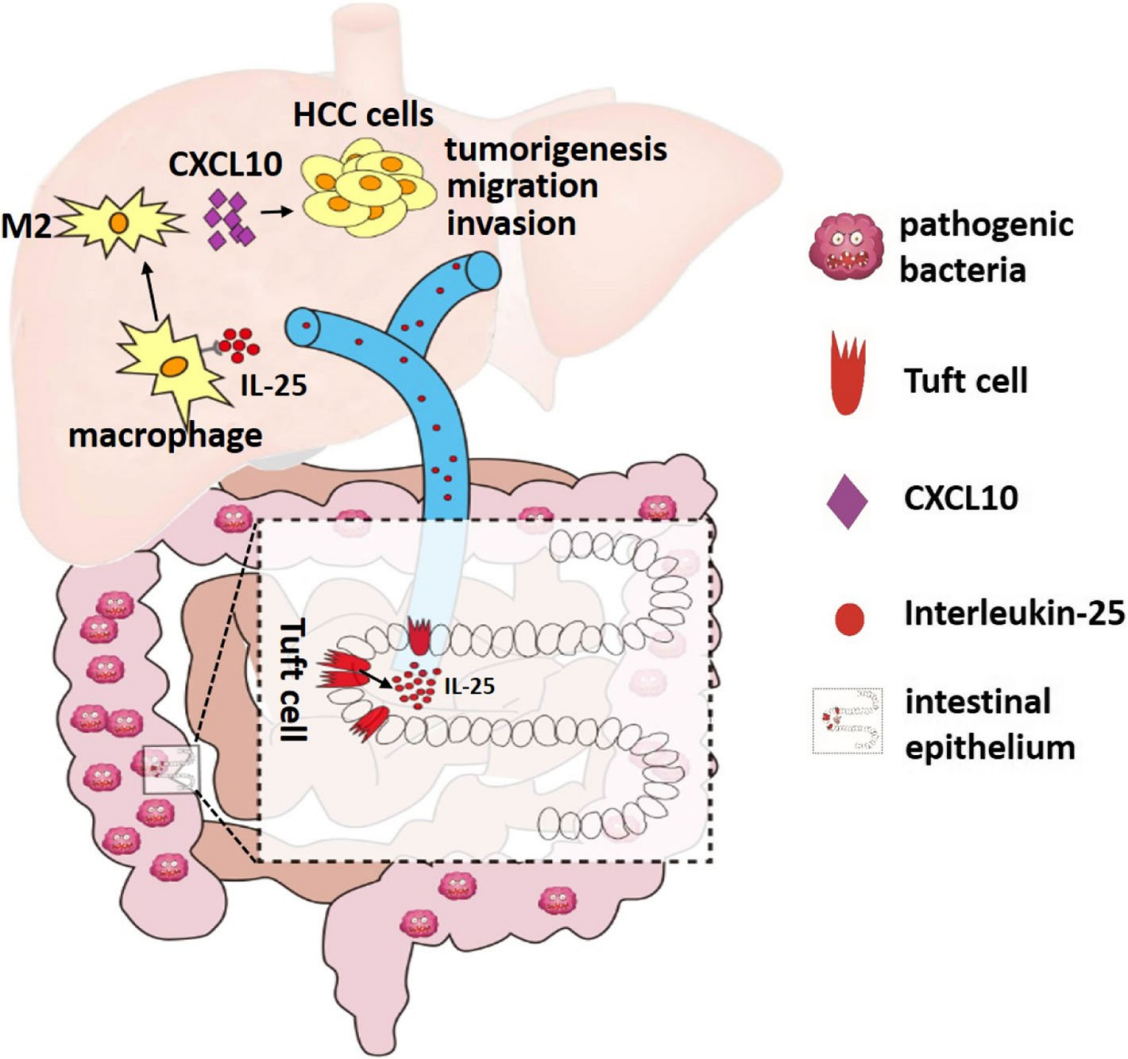
Gut bacterial dysbiosis results in colonic epithelial tuft cell hyperplasia and increased secretion of IL-25, which enters the liver via portal vein. IL-25 derived from the gut promotes the alternative activation of macrophages and fosters the tumorigenesis and migration of HCC cells via chemokine CXCL10

Our data is the first to demonstrate that IL-25 is associated with a poor prognosis in HCC patients, and can indirectly promote the progression of HCC. The mechanism by which IL-25 promotes HCC progression is that IL-25 induces the alternative activation of macrophages, which secrete the chemokine CXCL10 and activate the EMT pathway of HCC. Furthermore, gut microbiota dysbiosis can induce the hyperplasia of colonic epithelial tuft cells and the secretion of IL-25, which promotes HCC development (Fig. 6). Thus, our study not only identified the role of IL-25 in HCC, but also provides a potential new therapeutic target for HCC.

## Additional file

Additional file 1: **Table S1.** Clinical characteristic of human subjects. **Table S2.** Clinical characteristic of patients in survival analysis. **Table S3.** The sequence of primers. **Figure S1.** Direct treatment of HCC cells with IL-25 in vitro has no effect. (A) Cell growth determined by Cell Counting Kit-8 assay at 450 nm. (B) IL-25 did not induce MHCC97L and HepG2 cell lines apoptosis, as determined by Annexin V kit. (C) HCC cell growth determined by Brdu kit. Statistical data are shown at the right. Bar, 100 μm. (D) IL-25 had no impact on HCC cell migration, as determined by Transwell assay. Statistical data are shown at the right. Bar, 100 μm. (E) IL-25 did not significantly promote tumorigenesis of Hepa1-6 subcutaneous implanted cells in C57BL/6 mice. Statistical data are shown at the right. ns, no significance. **Figure S2.** M2 percentage (CD206/CD68) in HCC tumor tissues was negatively correlated with prognosis. (A, B) Immunohistochemistry staining was performed on a tissue microarray consisting of 98 HCC tumor tissues. Overall survival (A) and disease free survival (B) curves of HCC patients in correlation with intra-tumor M2 level (CD206/CD68). The third quartile of the M2 percentage was used as a cut-off value: low group (n = 70), high group (n = 28). The clinical characteristics of these two group are summarized in table S2. **Figure S3.** IL-25 facilitates chemokines secretion of macrophages. (A) Macrophages (derived THP-1) were treated with IL-25 and vehicle (negative control), respectively, for 48 h. The gene expression of chemokines was quantified by RT-qPCR. (B-C) Macrophages (derived THP-1) were treated with IL-25 in a time- and concentration- dependent manner. Gene expression of CXCL1, CCL2, CXCL10, and CCL17 was quantified by RT-qPCR. *p <0.05, **p <0.01, ***p <0.001, ns, no significance. (DOCX 502 kb)

## Data Availability

All data generated or analyzed during this study are included either in this article or in the supplementary information files.

## Abbreviations

ANMV: Combination antibiotics group
CCK-8: Cell Counting Kit-8
DFS: Disease-free survival
DMEM: Dulbecco’s modified Eagle’s medium
EMT: Epithelial-to-mesenchymal transition
ERK: Extracellular signal-regulated kinase
FBS: Fetal bovine serum
FH: Fulminant hepatitis
HCC: Hepatocellular carcinoma
HCV: Hepatitis C virus
HH: Hepatic hemangioma
HIV: Human immunodeficiency virus
IACUC: Institutional Animal Care and Use Committee
IL-25: Interleukin-25
M2: Type-2 macrophages
NAFLD: Nonalcoholic fatty liver disease
OS: Overall survival
PCoA: Principle coordinates analysis
PMA: Phorbol 12-myristate13-acetate
RT-qPCR: Real-time quantitative PCR
TAM: Tumor-associated macrophages
TME: Tumor microenvironment
WB: Western blotting
