# Supplementary material for: Intestinal dysbacteriosis-induced IL-25 promotes development of HCC via alternative activation of macrophages in tumor microenvironment
Source: J Exp Clin Cancer Res. 2019 Jul 11;38:303. doi: 10.1186/s13046-019-1271-3 (PMC6625119; doi:10.1186/s13046-019-1271-3)
Supplement: Supplementary file 1 — Table S1. Clinical characteristic of human subjects. Table S2. Clinical characteristic of patients in survival analysis. Table S3. The sequence of primers. Figure S1. Direct treatment of HCC cells with IL-25 in vitro has no effect. (A) Cell growth determined by Cell Counting Kit-8 assay at 450 nm. (B) IL-25 did not induce MHCC97L and HepG2 cell lines apoptosis, as determined by Annexin V kit. (C) HCC cell growth determined by Brdu kit. Statistical data are shown at the right. Bar, 100 μm. (D) IL-25 had no impact on HCC cell migration, as determined by Transwell assay. Statistical data are shown at the right. Bar, 100 μm. (E) IL-25 did not significantly promote tumorigenesis of Hepa1-6 subcutaneous implanted cells in C57BL/6 mice. Statistical data are shown at the right. ns, no significance. Figure S2. M2 percentage (CD206/CD68) in HCC tumor tissues was negatively correlated with prognosis. (A, B) Immunohistochemistry staining was performed on a tissue microarray consisting of 98 HCC tumor tissues. Overall survival (A) and disease free survival (B) curves of HCC patients in correlation with intra-tumor M2 level (CD206/CD68). The third quartile of the M2 percentage was used as a cut-off value: low group (n = 70), high group (n = 28). The clinical characteristics of these two group are summarized in table S2. Figure S3. IL-25 facilitates chemokines secretion of macrophages. (A) Macrophages (derived THP-1) were treated with IL-25 and vehicle (negative control), respectively, for 48 h. The gene expression of chemokines was quantified by RT-qPCR. (B-C) Macrophages (derived THP-1) were treated with IL-25 in a time- and concentration- dependent manner. Gene expression of CXCL1, CCL2, CXCL10, and CCL17 was quantified by RT-qPCR. *p <0.05, **p <0.01, ***p <0.001, ns, no significance. (DOCX 502 kb) [file 13046_2019_1271_MOESM1_ESM.docx]

**Materials and methods**

**Immunohistochemistry and immunofluorescence**

A tissue microarray was used to examine the level of IL-25 and number of M2 macrophages in normal liver tissue and in tumor tissue. The tissue sections were incubated with anti-IL-25 (Novus Biologicals, 1:400), anti-CD68 (Abcam, 1:400), anti-CD206 (Abcam, 1:2000), or anti-DCLK1(Abcam, 1:500) at 4^∘^C overnight. A Dako Real Envision Detection System (Dako) was then used for visualization. Images were obtained with an Olympus BX63 microscope and ZEISS Axio Scan.Z1 Digital Slide Scanner. The immunohistochemical staining in the tissue was scored independently by 2 pathologists blinded to the clinical data.

Immunofluorescence staining was performed on cells embedded in Millicell EZ slides (Millipore), and on frozen sections. Double-immune fluorescence staining was performed using anti-DCLK1 (Abcam, 1:1000), anti-IL-25 (Santa Cruz Biotechnology, 1:50 ), anti-CD68 (Abcam, 1:200), and anti-CD206 (Abcam, 1:1000) as the primary antibody, and Alex Fluor 594-donkey anti-rabbit IgG, Alex Fluor 488-donkey anti-goat IgG, Alex Fluor 594-donkey anti-mouse IgG, and Alex Fluor 488-donkey anti-rabbit IgG (Life Technologies, 1:200) as the secondary antibody, respectively. The tissue slices were digitally photographed with a confocal microscope.

**Migration and invasion transwell assays**

Migration and invasion transwell assays were performed in a 24-well cell culture chamber using inserts with 8 μm sized pores (Corning). MHCC97L cells (3 × 10^5^/well) or HepG2 cells (2 × 10^5^/well) were cultured in the top chamber of 24-well Transwell plates with (for invasion assay) or without (for migration assay) Matrigel (BD Biosciences). Inserts containing MHCC97L or HepG2 cells were transferred to wells containing 2 × 10^5^ M2 polarized THP-1 cells for 24 h and 36 h co-culture for migration and invasion assays, respectively. To test whether IL-25 could directly induce migration and invasion of HCC cells, 100 ng/ml of recombinant human IL-25 was added to each well instead of macrophages. The migrated cells were stained with crystal violet, and the numbers of migrated cells were determined by microscopy. Five fields (100 × magnification) were randomly selected, the numbers of invaded cells were counted, and the average number of invaded cells was calculated.

**Cell** **proliferation and apoptosis assays**

HCC cells were treated with IL-25 (Biolegend) or co-cultured with M2 cells for cell growth and apoptosis assays, respectively. Cell proliferation was detected by a Cell Counting Kit-8 (CCK-8) (Dojindo, Japan) or Brdu staining (Sigma-Aldrich) according to the manufacturer’s protocol. Cell apoptosis was detected using an FITC Annexin V apoptosis detection kit according to the manufacturer’s protocol (BD Biosciences). The cell death percentage was defined as the ratio of annexin V(+)/PI(+) stained cells times 100. The apoptotic cell death percentage was defined as the ratio of annexin V(+)/PI(−) stained cells times 100.

**Quantitative real time RT-PCR**

Total RNA was extracted from cells using TRIzol reagent (Invitrogen). Complementary DNA (cDNA) was synthesized using the Prime Script RT Reagent Kit Perfect Real-Time Kit (TaKaRa Bio Inc., Japan). The cDNA was then used for quantitative real-time PCR (RT-qPCR) using SYBR PremixEx Taq (TaKaRa Bio Inc.). The detail experimental procedure was according to the manufacturer’s instructions respectively. The relative expression levels of mRNAs were calculated by the 2-ddCt method.

**ELISA and Western blotting**

Blood samples were centrifuged at 1000 *g* for 20 min. Tissue samples were homogenized in ice-cold PBS (for ELISA) or protein lysis buffer (for Western blotting) with protease inhibitor cocktail, followed by centrifugation at 1500 *g* for 10 min and the supernatants were collected. Cells were harvested and lysed for Western blotting. Protein concentration was measured using the KeyGen Protein Assay Kit (KeyGen). IL-25 protein in serum and tissue was detected using an ELISA kit (Cloud-Clone Corp.) according to the manufacturer’s instructions. Anti-IL-25 (Novus Biologicals, #NB100-56541, 1:1000), anti-DCLK1, anti-CXCL10, anti-CD206 (Abcam, 1:1000), anti-TNF-α, anti-E-cadherin, anti-vimentin, anti-Snail (Cell Signaling Technology, 1:1000), anti-ERK, anti-p-ERK (Santa Cruz Biotechnology, 1:1000) and anti-β-actin (Sigma-Aldrich, 1:10000) were used in the Western blotting assays.

**Subcutaneous implanted tumor model**

All studies were conducted with the approval of the Institutional Animal Care and Use Committee (IACUC) of the First Affiliated Hospital of Sun Yat-Sen University. Male C57BL/6 mice (4-6 weeks old) were purchased from Vital River Laboratories (Beijing, China). The mice were randomly divided into a control group (n = 5) and an IL-25 treatment group (n = 5). Mouse HCC cell line Hepa1-6 cells (5 × 10^6^ in 200 µl DMEM) were implanted subcutaneously into the right flank of each mouse. The mice were then treated with recombinant mouse IL-25 (1,000 ng in 200 µl per mouse) or vehicle by intraperitoneal injection 2 times a week. All mice were killed at the fourth week, and the weight of liver tumor tissue was measured.

**Orthotopic nude mice liver tumor model**

Male BALB/c nude mice (4-6 weeks old) were purchased from Vital River Laboratories (Beijing, China). The mice were randomly divided into a control group (M0 treatment group, n = 8) and an M2 macrophage treatment group (n = 8). Operative procedures were as described previously. Briefly, 2 × 10^6^ MHCC97L cells suspended in 200 µl DMEM were injected subcutaneously into the flanks of the mice. After 4 weeks, the subcutaneous tumors were resected and diced into 1 mm^3^ cubes, which were then implanted in the left lobes of the livers of the mice. Simultaneously, 5 × 10^5^ M0 or M2 polarized THP-1 macrophages suspended in 200 µl DMEM were injected into the portal vein of the same group of mice. All mice were killed at the sixth week, and the size of liver tumors was measured.

**Orthotopic C57BL/6 mice hepatic tumor model with gut microflora dysbiosis**

Male C57BL/6 mice (6-8 weeks old) were purchased from Vital River Laboratories (Beijing, China). The mice were randomly divided into 4 groups (n = 10 each group). Gut-sterilization was done as previously described using a combination of ampicillin (1 g/l), neomycin (1 g/l), metronidazole (1 g/l), and vancomycin (500 mg/l) in drinking water for 4 weeks (AVMV group) . Gut bacterial dysbiosis was induced using vancomycin (mainly sterilizes Gram-positive bacteria, 500 mg/l) or cefoperazone (mainly sterilizes Gram-negative bacteria, 400 mg/l) in drinking water for 4 weeks. Mice in the control group were given sterile water with nothing added. H22 cells (1 × 10^6^ in 200 µl DMEM) were injected subcutaneously into the flanks of the C57BL/6 mice. After 2 weeks, the subcutaneous tumors were resected and diced into 1 mm^3^ cubes, which were then implanted in the left lobes of the livers of each group of C57BL/6 mice with continuing antibiotic treatment. Two mice in the vancomycin group and 1 in the cefoperazone group died during the surgery. All mice were killed after 2 weeks, the size of the liver tumors was measured, and samples were collected for the next study.

Table S1. Clinical characteristic of human subjects

| Sample characteristics | Serum/Tissue | | Tissue microarray | |
| --- | --- | --- | --- | --- |
|  | HH | HCC | HH | HCC |
| Number of patients | 5 | 10 | 55 | 98 |
| Age (yr, median, range) | 36, 26-43 | 49, 29-64 | 45, 27-85 | 48, 23-75 |
| Gender (male/female) | 1/4 | 7/3 | 21/34 | 92/6 |
| HBsAg (negative/positive) | 5/0 | 1/9 | 50/5 | 14/84 |
| Cirrhosis (absent/present) | 5/0 | 6/4 | 55/0 | 15/83 |
| ALT (U/L, ≤40/>40) | 5/0 | 5/5 | 55/0 | 37/61 |
| AFP (ng/ml, ≤20/>20) | 5/0 | 3/7 | 55/0 | 17/75 |
| Child-Pugh (A/B/C) | 5/0/0 | 10/0/0 | 55/0 | 88/10/0 |
| Tumor size (cm, ≤5/>5) | 0/5 | 5/5 | 0/55 | 34/64 |
| Number of tumor (single/more) | 1/4 | 6/4 | 9/46 | 62/36 |
| Vascular invasion (absent/present) | -/- | 6/4 | -/- | 65/33 |
| TNM stage (Ⅰ+Ⅱ/Ⅲ+Ⅳ) | -/- | 5/5 | -/- | 47/51 |

HH, hepatic hemangioma. HCC, hepatocellular carcinoma. HBsAg, hepatitis B surface antigen. ALT, alanine aminotransferase. AFP, a-fetoprotein. TNM, tumor node metastasis.

Table S2. Clinical characteristic of patients in survival analysis

| Sample characteristics | IL-25 | | M2 | |
| --- | --- | --- | --- | --- |
|  | Low | High | Low | High |
| Number of patients | 70 | 28 | 70 | 28 |
| Age (yr, median, range) | 48, 23-75 | 48, 26-75 | 48, 29-75 | 49, 23-75 |
| Gender (male/female) | 65/5 | 27/1 | 65/5 | 27/1 |
| HBsAg (negative/positive) | 7/63 | 4/24 | 6/64 | 5/23 |
| Cirrhosis (absent/present) | 10/60 | 4/24 | 10/60 | 4/24 |
| ALT (U/L, ≤40/>40) | 28/42 | 9/19 | 27/43 | 10/18 |
| AFP (ng/ml, ≤20/>20) | 10/60 | 7/21 | 8/62 | 9/19 |
| Child-Pugh (A/B/C) | 61/9/0 | 27/1/0 | 61/9/0 | 27/1/0 |
| Tumor size (cm, ≤5/>5) | 25/45 | 9/19 | 22/48 | 12/16 |
| Number of tumor (single/more) | 47/23 | 15/13 | 43/27 | 19/9 |
| Vascular invasion (absent/present) | 49/21 | 16/12 | 47/23 | 8/10 |
| TNM stage (Ⅰ+Ⅱ/Ⅲ+Ⅳ) | 35/35 | 10/18 | 32/38 | 13/15 |

HH, hepatic hemangioma. HCC, hepatocellular carcinoma. HBsAg, hepatitis B surface antigen. ALT, alanine aminotransferase. AFP, a-fetoprotein. TNM, tumor node metastasis.

Table S3. The sequence of primers

| Gene | Forward 5’-3’ | Reverse 5’-3’ |
| --- | --- | --- |
| CXCL1 | AACCGAAGTCATAGCCACAC | GTTGGATTTGTCACTGTTCAGC |
| CCL2 | CCCAAAGAAGCTGTGATCTTCA | TCTGGGGAAAGCTAGGGGAA |
| CCL5 | CAGTCGTCCACAGGTCAAGG | CTTGTTCAGCCGGGAGTCAT |
| CXCL8 | CACCGGAAGGAACCATCTCA | TGGCAAAACTGCACCTTCACA |
| CXCL9 | AGTGCAAGGAACCCCAGTAG | AGGGCTTGGGGCAAATTGTT |
| CXCL10 | CCACGTGTTGAGATCATTGCT | TGCATCGATTTTGCTCCCCT |
| CXCL12 | AGATTGTAGCCCGGCTGAAG | CAGGCCCTTCCCTAACACTG |
| CCL15 | CAAGCCAGGTGTCATATTCCTC | AACTCACAGGAGGTGTTGGA |
| CCL19 | GGTGCCTGCTGTAGTGTTCA | TGCAGTCTCTGGATGATGCG |
| CCL20 | CGAATCAGAAGCAGCAAGCAA | TTGCGCACACAGACAACTTT |
| CX3CL1 | CTCCGATATCTCTGTCGTGGC | TGTCTCGTCTCCAAGCAGC |
| CCL17 | GAGCCATTCCCCTTAGAAAG | AGGCTTCAAGACCTCTCAAG |
| CCL22 | AGGACAGAGCATGGATCGCCTACAGA | TAATGGCAGGGAGGTAGGGCTCCTGA |
| β-actin | GCACTCTTCCAGCCTTCCTT | GTTGGCGTACAGGTCTTTGC |


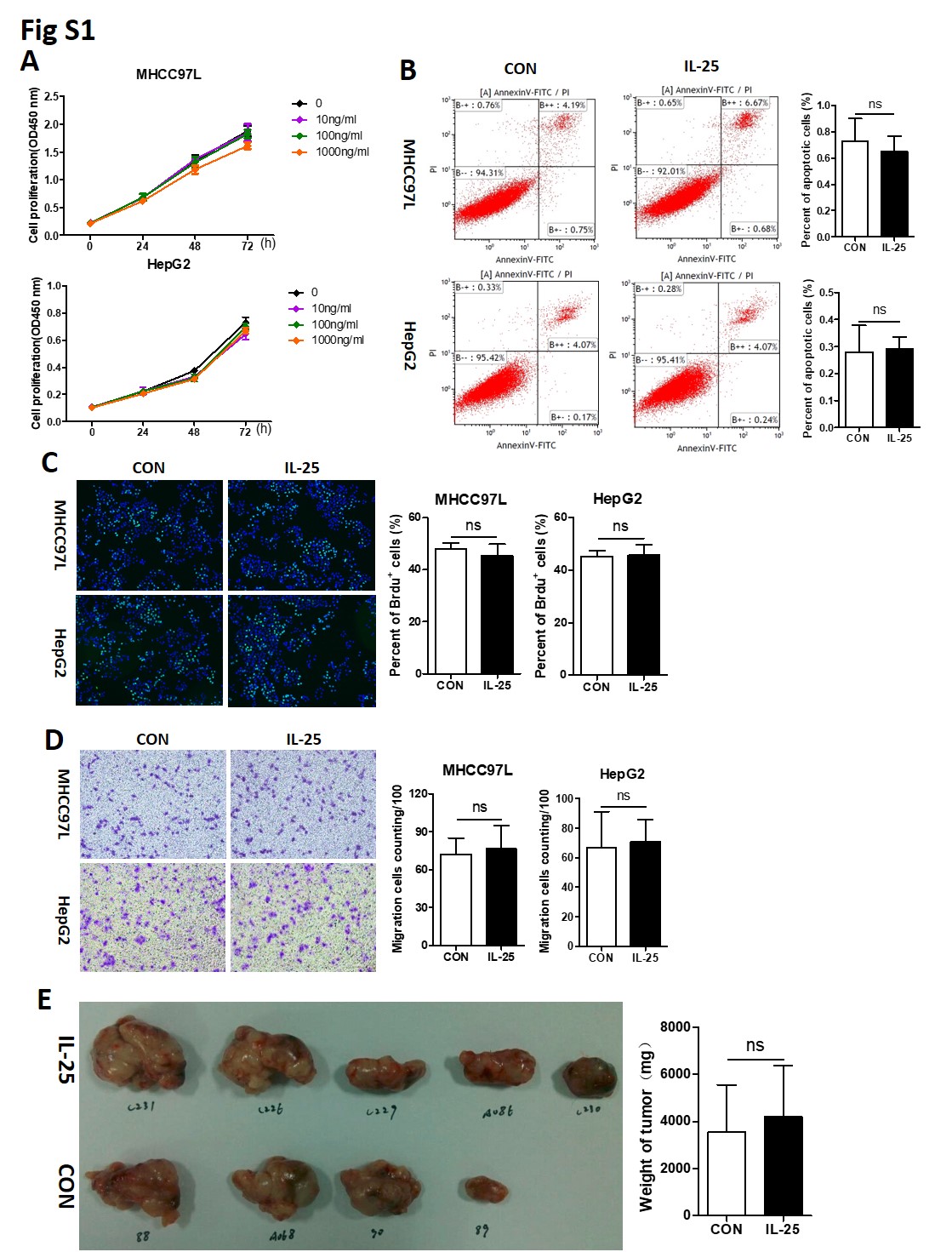


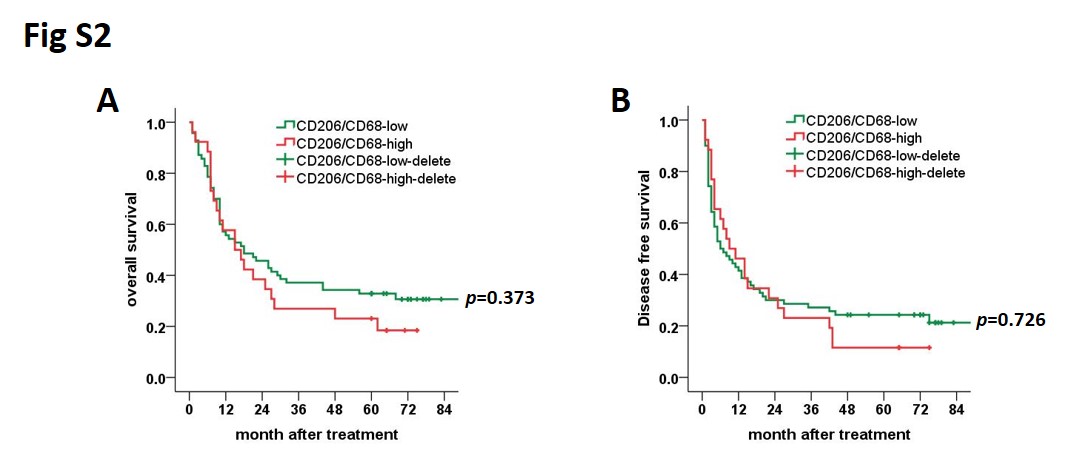


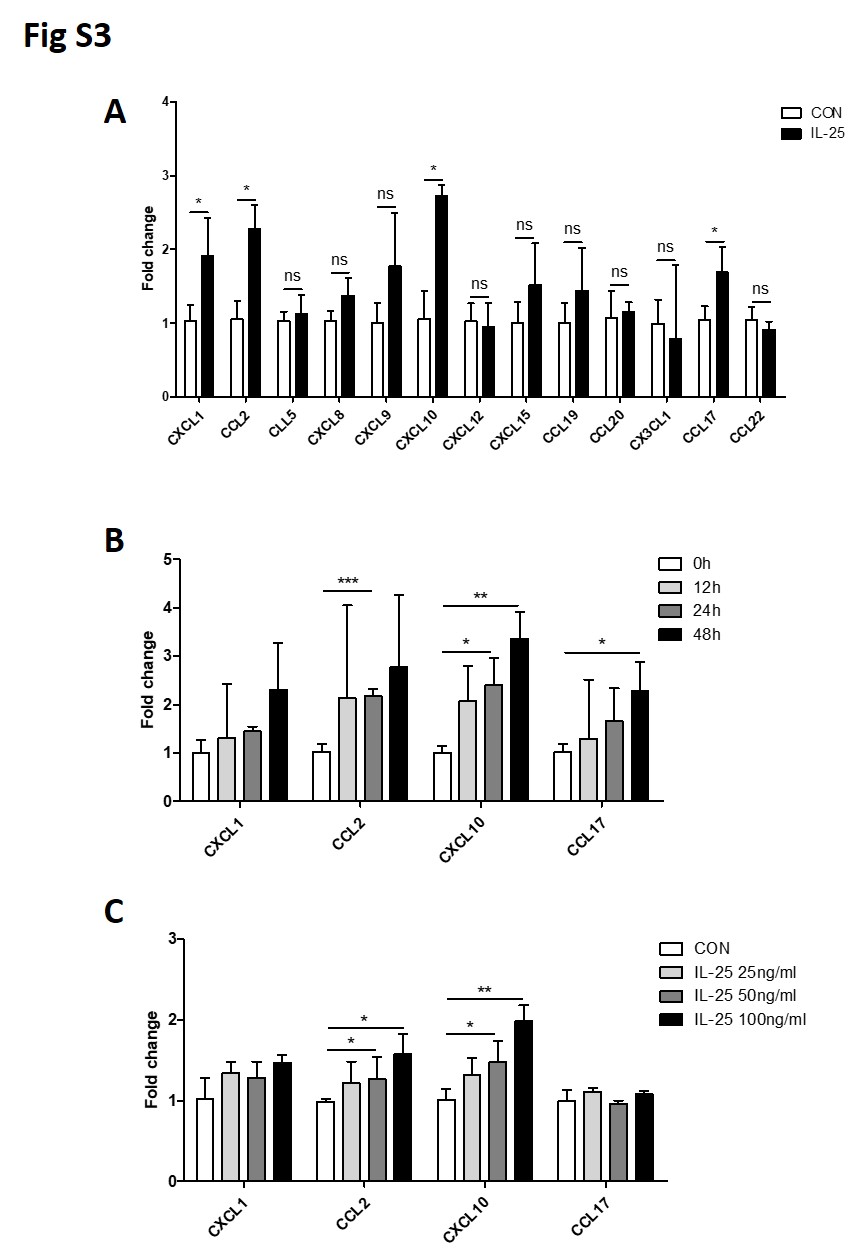


**Figure Legends**

**Figure S1. Direct treatment of HCC cells with IL-25 *in vitro* has no effect.**

(A) Cell growth determined by Cell Counting Kit-8 assay at 450 nm.

(B) IL-25 did not induce MHCC97L and HepG2 cell lines apoptosis, as determined by Annexin V kit.

(C) HCC cell growth determined by Brdu kit. Statistical data are shown at the right. Bar, 100 µm.

(D) IL-25 had no impact on HCC cell migration, as determined by Transwell assay. Statistical data are shown at the right. Bar, 100 µm.

(E) IL-25 did not significantly promote tumorigenesis of Hepa1-6 subcutaneous implanted cells in C57BL/6 mice. Statistical data are shown at the right.

ns, no significance.

**Figure S2. M2 percentage (CD206/CD68) in HCC tumor tissues was negatively correlated with prognosis.**

(A, B) Immunohistochemistry staining was performed on a tissue microarray consisting of 98 HCC tumor tissues. Overall survival (A) and disease free survival (B) curves of HCC patients in correlation with intra-tumor M2 level (CD206/CD68). The third quartile of the M2 percentage was used as a cut-off value: low group (n = 70), high group (n = 28). The clinical characteristics of these two group are summarized in online supplementary table S2.

**Figure S3. IL-25 facilitates chemokines secretion of macrophages.**

(A) Macrophages (derived THP-1) were treated with IL-25 and vehicle (negative control), respectively, for 48 h. The gene expression of chemokines was quantified by RT-qPCR.

(B-C) Macrophages (derived THP-1) were treated with IL-25 in a time- and concentration- dependent manner. Gene expression of CXCL1, CCL2, CXCL10, and CCL17 was quantified by RT-qPCR.

**p* < 0.05, ***p* < 0.01, ****p* < 0.001, ns, no significance.
